# Postoperative Dehydration Is Associated with Frailty and Decreased Survival in Older Patients with Hip Fracture

**DOI:** 10.3390/nu14040820

**Published:** 2022-02-16

**Authors:** Michela Zanetti, Paolo De Colle, Cinzia Omiciuolo, Chiara Ratti, Gianluca Gortan Cappellari, Rocco Barazzoni, Luigi Murena, Gianfranco Sanson

**Affiliations:** 1 Clinica Medica, Clinical Department of Medical, Surgical and Health Sciences, University Hospital of Trieste, Strada di Fiume 447, 34100 Trieste, Italy; barazzon@units.it; 2 Geriatria, Department of Medicine, University Hospital of Trieste, Piazza dell’Ospitale 1, 34100 Trieste, Italy; paolo.decolle@asugi.sanita.fvg.it (P.D.C.); cinzia.omiciuolo@asugi.sanita.fvg.it (C.O.); 3 Clinica Ortopedica, Clinical Department of Medical, Surgical and Health Sciences, University Hospital of Trieste, Strada di Fiume 447, 34100 Trieste, Italy; chiara.ratti@asugi.sanita.fvg.it (C.R.); lmurena@units.it (L.M.); 4 Clinical Department of Medical, Surgical and Health Sciences, University Hospital of Trieste, Strada di Fiume 447, 34100 Trieste, Italy; ggortancappellari@units.it (G.G.C.); gsanson@units.it (G.S.)

**Keywords:** dehydration, frailty, hip fracture, older, orthogeriatric care

## Abstract

Background: Hyperosmolar dehydration (HD) is a risk factor for severe complications in hip fracture in older patients. However, evidence for recommending screening of dehydration is insufficient and its relation with frailty and mortality is unclear. We tested the hypothesis that postoperative HD is associated with frailty and increased mortality. Methods: We recruited 625 older (>65 years) patients surgically treated for hip fracture and co-managed by an orthogeriatric team over one year in 2017. Pre- and postoperative HD (serum osmolarity > 300 mmol/L) was diagnosed. Frailty and associated mortality risk were assessed by the Multidimensional Prognostic Index (MPI). Results: The prevalence of preoperative HD was 20.4%. Compared with no-HD, MPI was similar in HD patients despite higher (*p* < 0.05) prevalence of polypharmacy, arterial hypertension, diabetes, chronic kidney disease and heart failure. After surgery the incidence of HD decreased to 16.5%, but increased (*p* = 0.003) in the MPI high-risk subgroup. Postoperative HD was associated with more complications and was an independent determinant of adjusted hospital length of stay (LOS) and of 60- to 365-days mortality. Conclusions: Older frail patients with hip fracture are prone to developing postoperative HD, which independently predicts prolonged hospital LOS and mortality. Systematically screening older patients for frailty and dehydration is advisable to customize hydration management in high-risk individuals.

## 1. Introduction

Preoperative dehydration is a well-recognized, potentially modifiable prognostic factor of in-hospital complications, hospital readmissions and of poor functional outcomes in older patients with hip fracture [1,2,3]. Previous studies have suggested that up to 50% of patients show signs of dehydration before and after hip fracture surgery [4,5,6]. Multiple factors potentially contribute to this condition, including increased urea production due to fracture-related bleeding and pre-renal azotemia secondary to insufficient fluid intake [7], as well as—most importantly—impaired renal capacity to preserve water and to govern the hemodynamic balance of fluids, a derangement typically associated with hemodynamic frailty [8].

In addition to dehydration, several modifiable and non-modifiable risk factors influence morbidity, mortality and functional recovery after hip fracture [2,3,9,10], which include surgical variables, as well as pre-fracture patient demographics, social, functional and medical conditions. The combination of the latter factors is best described by the frailty syndrome, which is notably characterized by a decline in multiple domains and by the presence of comorbidities and polypharmacy [11]. By ranking hip fracture patients according to frailty, it has been demonstrated that about 30% of them are frail [12] and they are predisposed to adverse clinical outcomes, including postoperative mortality, complications and prolonged length of hospital stay LOS [12,13]. Therefore, a standardized approach based on perioperative risk evaluation and treatment optimization, as in the integrated orthogeriatric care management, is mandatory, since it results in improved short- and long-term clinical outcomes, including morbidity, LOS, functional recovery and mortality [14,15,16,17].

Although clinical frailty scoring tools usually explore several domains, it is not clear whether in older patients with hip fracture the occurrence of pre- and postoperative dehydration can be predicted on the basis of the clinical frailty assessment performed at admission. In addition, the relative roles of pre- and postoperative dehydration on the burden of morbidity and mortality in these patients has been scarcely explored. Based on available literature we may hypothesize that frailer subjects are more prone to dehydration, which contributes to adverse clinical outcomes.

Therefore, the aims of this study were: (1) to describe the prevalence of postoperative dehydration as compared to the corresponding preoperative value in a cohort of patients acutely admitted for hip fracture; (2) to assess its associations with frailty and (3) its relationships with postoperative complications, length of hospital stay and short- medium- and long-term mortality.

## 2. Materials and Methods

### 2.1. Study Design, Setting and Population

A retrospective observational study was conducted in the University Hospital of Trieste, Italy. All consecutive patients aged ≥ 65 years admitted because of a femur fracture from 1 January to 31 December 2017 and in the charge of the orthogeriatric team (in which patients were co-managed by orthopedics and geriatricians) were considered for eligibility. Patients undergoing conservative treatment (no surgery) and those with incomplete laboratory data to compute preoperative serum osmolarity were excluded. 

### 2.2. Study Variables

Clinical, functional and serum biochemistry data assessed during the hospital stay were collected from the patients’ electronic medical records. Sociodemographic variables were also collected and two age categories (≤85 and >85 years) were created. Moreover, the chronic use of medications potentially affecting the hydration status was documented.

The Krahn and Khajuria equation (osmolarity = 1.86 × (Na^+^ + K^+^) + 1.15 × glucose + urea + 14; each component measured in mmol/L) [18], was used to calculate serum osmolarity. This equation has been validated in very different populations of older adults [19] and has demonstrated to be an accurate and objective diagnostic tool to assess hyperosmolar dehydration in older hospitalized medical patients [20]. Patients with calculated osmolarity of > 300 mmol/L were considered as having a hyperosmolar dehydration (HD) [21]. 

The eGFR was calculated through the equation developed by Levey et al. [22]. 

Severity of frailty was scored according to the Multidimensional Prognostic Index (MPI), an assessment tool including information on functional, nutritional, cognitive and social status, polypharmacy and comorbidities, which has been developed for hospitalized older subjects and has proved to predict short- and long-term adverse clinical outcomes including mortality [11,23,24]. The MPI was computed upon hospital admission based on the routinely collected Comprehensive Geriatric Assessment (CGA), establishing three risk levels: low (0.0–0.33); moderate (0.34–0.66); and severe (0.67–1.00) [24]. 

The Glasgow Prognostic Score (GPS) was adopted as a cumulative inflammation-based score, reported as a reliable prognostic marker in acutely hospitalized patients [25]. The GPS was calculated based on the combination of albumin and C-reactive protein levels, identifying the following categories: 0 (good prognosis—CRP ≤ 10 mg/L and albumin ≥ 3.5 g/dL); 1 (intermediate prognosis—CRP > 10 mg/L and albumin ≥ 3.5 g/dL, or CRP ≤ 10 mg/L and albumin < 3.5 g/dL); and 2 (poor prognosis—CRP > 10 mg/L and albumin < 3.5 g/dL). 

Both osmolarity and eGFR were computed pre- (the day before surgery) and postoperatively (the day after surgery), while GPS was available only in the preoperative phase since serum albumin was determined just once, upon patient admission.

### 2.3. Study End-Points

The primary study endpoint was all-cause mortality—defined as death from any cause within 12-months from hospital admission. Date of death was obtained from the electronic mortality register. Moreover, the possible independent association between postoperative HD and total hospital length of stay (LOS) was explored.

As a secondary endpoint, the association of postoperative HD with the occurrence of the following postoperative complications (defined according to combinations of clinical, laboratory and instrumental data) was documented: acute respiratory failure, pneumonia, delirium, blood glucose imbalance (i.e., occurrence of hyperglycemia) or electrolyte imbalance (i.e., occurrence of hyper-hyponatremia or hyper-hypokalemia), pressure ulcers, urinary tract infections, heart failure exacerbation, surgical wound infection and total number of transfused blood units. Finally, the association between the level of frailty and the modification of serum osmolarity between pre- and postoperative stages was explored.

### 2.4. Data Analysis

Since we recruited a convenience sample including all consecutive patients over one year, no formal sample size was calculated a priori. The number of deaths observed in the study population was sufficient to satisfy the pragmatic thumb rule of 1:10 predictors to events ratio in the most multivariable models. 

Data distribution was evaluated using the Kolmogorov–Smirnov test. The continuous variables were presented as medians and interquartile ranges (IQRs). Unadjusted comparisons between the groups were analyzed via nonparametric Mann–Whitney’s U test for independent samples or Wilcoxon test for paired samples, as appropriate. Nominal variables were shown as a number and percentage and were analyzed using contingency tables and χ test or Fisher’s exact test, as appropriate.

The independent association between a postoperative HD condition and the patients’ hospital LOS was investigated by forward stepwise multiple linear regression model, after controlling for study variables associated to the hospital LOS with a *p*-value of <0.1. Since the hospital LOS data had a skewed distribution, logarithmic transformation was performed to obtain a more approximately normal variable. Results were reported as *β* and *p* values.

The different time of death according to the occurrence of postoperative HD was explored. Observations were right-censored until 30, 60, 90, 180 or 365 days after hospital admission (i.e., known survival). Both unadjusted and adjusted analyses were performed. Crude evaluation was carried out by comparing Kaplan–Meier curves and differences in survival rates between groups were assessed with Log-rank Mantel-Cox test and presented with crude Kaplan–Meier curves. Adjusted comparisons were performed by fitting multivariable Cox proportional-hazards models with forward stepwise selection, adjusting for confounders potentially affecting serum osmolarity (i.e., eGFR) or known to be related to mortality in older acute inpatients. The results were presented as an adjusted proportional hazard ratio (HR) and 95% confidence intervals (CI); cumulative hazard adjusted curves were drawn, as well. 

All statistical analyses were performed using the software IBM SPSS Statistics, version 24.0 (IBM Corp., Armonk, NY, USA) For all tests, an alpha level of *p* ≤ 0.05 was set for statistical significance.

## 3. Results

**Figure 1:**
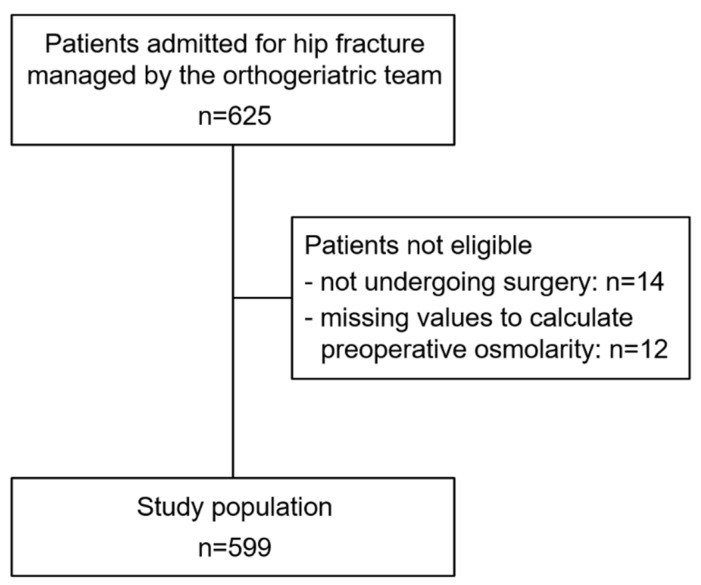
Flow diagram of the participants’ selection process.

During the study period, 625 older patients were admitted to the hospital and managed by the orthogeriatric team because of a hip fracture. After excluding subjects not undergoing surgery and/or having insufficient data to compute preoperative serum osmolarity, 599 patients were included in the study population (Figure 1).

Table 1 shows the main characteristics of the patients. They were mostly females (74.8%), of median age 84 years old. Median MPI value was 0.50 with 23.5%, 53.3% and 22.7% of patients in the low, moderate and severe risk groups, respectively. The most represented comorbidity was arterial hypertension. Just under half of the patients were on polypharmacy with the most prescribed drugs represented by anti-hypertensives, neuroleptics and benzodiazepines, and diuretics. Among patients chronically assuming diuretics, the most frequently prescribed medications were furosemide (*n* = 127, 66.8%)—alone (*n* = 92, 72.4%) or associated with other diuretics (spironolactone: 27, 21.3%; other diuretics: 8, 6.3%), and hydrochlorothiazide (alone: 48, 25.3%; with amiloride: 10, 5.3%). Among laboratory parameters, the median-calculated serum osmolarity was 295 mmol/L with a prevalence of preoperative hyperosmolar dehydration (HD) of 20.4% (*n* = 122). 

Compared with the no-HD subgroup, HD patients showed a higher (*p* < 0.05) BMI, a greater prevalence of arterial hypertension, chronic kidney disease and heart failure and diabetes, with an increased percentage of polypharmacy and a higher rate of home therapy with diuretics. Moreover, HD patients presented preoperatively with poorer eGFR, lower haemoglobin levels and higher concentrations of serum urea, sodium, potassium and glucose exposing to a HD condition as represented by higher serum osmolarity. Conversely, the two subgroups did not differ in age, sex, living condition and MPI, as well as in the other considered comorbid and laboratory variables.

Patients underwent surgery after a median time of 2.0 (IQR 1.0–3.0) days from admission. Data to compute postoperative osmolarity were available for 595 patients (99.3%). Overall, a statistically significant reduction in serum osmolarity was found after surgery (pre: 295.0, IQR 290.5–299.3 mmol/L; post: 293.4, IQR 288.8–297.7 mmol/L; *p* < 0.001), the incidence of postoperative HD being 16.5% (*n* = 99). The incidence of postoperative HD was higher in patients with chronic heart failure (no-HD: *n* = 63, 12.7%; HD: *n* = 30, 30.3%; *p* < 0.001), hypertensive (no-HD: *n* = 94, 19.0%; HD: *n* = 29, 29.3%; *p* = 0.020) or ischemic heart disease (no-HD: *n* = 83, 16.7%; HD: *n* = 29, 29.3%; *p* = 0.004), and chronic kidney disease (no-HD: *n* = 42, 8.5%; HD: *n* = 32, 32.3%; *p* < 0.001). Table 2 describes the comparisons between patients presenting or not a postoperative HD.

**Figure 2:**
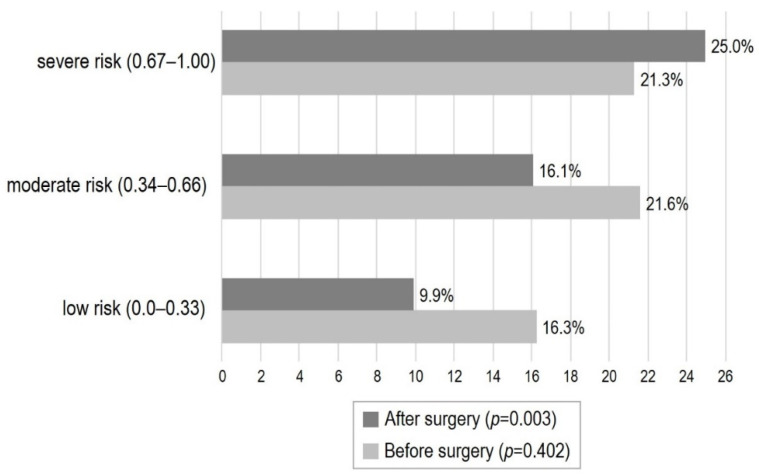
Modification in HD prevalence before and after surgery according to MPI groups.

No between-group difference was found according to the type of surgery. A statistically significant higher HD incidence was documented among patients who underwent surgery after two or more days from hospital admission. Overall, 259 patients (43.2%) underwent blood transfusions; a more frequent need for transfusions was documented among patients with postoperative HD. When considering the occurrence of complications, no patient presented a surgical wound infection. Individuals with postoperative HD showed a higher incidence of pneumonia, electrolyte imbalance, pressure ulcers and exacerbation of heart failure, while only a non-significant trend was documented toward a higher incidence of respiratory failure. No further statistically significant difference was documented when considering the association of postoperative HD with the other complications. After stratifying the study population according to MPI risk categories, in postoperative stage the HD prevalence decreased in low- and moderate-risk groups, and it increased among patients scored as MPI high-risk (Figure 2), with a statistically significant difference after surgery (*p* = 0.003).

After excluding patients deceased before hospital discharge (*n* = 31), the median hospital LOS of the study population was 12.0 days (IQR 9.0–15.0). Hospital LOS was higher (*p* = 0.005) in females (12.0 days, IQR 9.0–15.0 vs. 13.0 days, IQR 10.0–16.0 in males), in patients with postoperative dehydration (no-HD: 12.0 days, IQR 9.0–15.0; HD: 13.0 days, IQR 11.0–16.0, *p* = 0.001), and in those with increased GPS (GPS 0–1: 12.0 days, IQR 9.0–15.0; GPS 2: 13.0 days, IQR 10.0–16.0; *p* = 0.019), while no statistically significant difference was detected for MPI (*p* = 0.055), age (*p* = 0.125), and postoperative eGFR (*p* = 0.099). Postoperative HD was a statistically significant independent determinant of hospital LOS in multiple linear regression analysis adjusted for sex, MPI, GPS and postoperative eGFR (Table 3).

**Figure 3:**
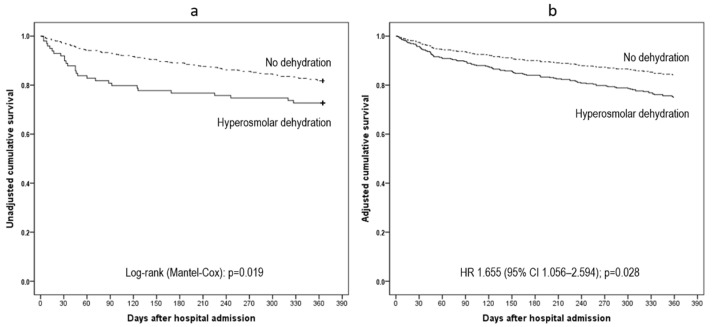
Crude (**a**) and adjusted (**b**) Kaplan–Meier one-year survival curves for patients with or without postoperative hyperosmolar dehydration.

All-cause mortality data within 12 months from hospital admission were available in 99.5% of cases (*n* = 596). The cumulative mortality rate was 4.0%, 8.1%, 9.1%, 13.3%, and 20.0% at 30, 60, 90, 180, and 365 days, respectively. Results of unadjusted and adjusted survival analyses are reported in Table 4; crude and adjusted 365 days survival curves are shown in Figure 3. In unadjusted analyses, the crude risk of death in all considered time intervals for patients with postoperative HD was significantly higher than that of those without this condition (Table 4).

All variables considered to adjust the regression models (i.e., age > 85, male sex, MPI-severe risk, and GPS-poor prognosis) showed a statistically significant association (*p* < 0.05) with one-year mortality, except for eGFR (*p* = 0.089). In multivariable analyses, patients with postoperative HD showed a statistically significant increased risk of death at 60- to 365 days, while the same predictive power was not confirmed for 30 days mortality. 

## 4. Discussion

In this retrospective observational study including a large cohort of older patients who underwent surgery for hip fracture we found that: (1) preoperative dehydration is associated with comorbidities and with polypharmacy but not with frailty; (2) in the postoperative phase the prevalence of dehydration is reduced in patients at low- and moderate-risk MPI categories, while it markedly increases in the high-risk MPI subgroup; (3) postoperative dehydration predisposes the patients to complications including pneumonia and acute heart failure; and (4) it is independently associated with increased length of hospital stay and with mortality in the medium- and long-term. 

Screening for dehydration in the perioperative phase is not routine clinical practice. The issue is relevant, because 30–50% of older hip fracture patients preoperatively are affected by this condition [5,6] which has a direct negative impact on postoperative complications [6] and on rehabilitation [2,3]. In contrast, an adequate fluid management targeted at achieving hemodynamic stability results in a significant reduction of morbidity and mortality rates [26]. The diagnosis of dehydration is challenging, because most of clinical signs and laboratory tests are notably biased by low diagnostic accuracy [27]; however, at present plasma osmolarity is considered the most valuable parameter to assess hydration status and is currently recommended to identify dehydration in older patients [20,21]. By using serum osmolarity we found that 20.4% of study patients were dehydrated in the preoperative phase. This finding is in line with that reported by Loffel et al. [28] who used a composite score based on urine and blood tests to diagnose dehydration in older patients undergoing urological surgery while another study conducted a in hip fracture cohort reported a higher prevalence [6]. In addition, consistent with available literature, these findings demonstrate that some comorbidities, including renal failure and diabetes, are associated with dehydration in older people as well as polypharmacy and the use of diuretics [29,30]. In this study, preoperative dehydration was not related to frailty measured by the Multidimensional Prognostic Index which includes information on nutritional, functional, cognitive and social status as well as on polypharmacy and comorbidities [24]. This is in line with previous data from our group in a different cohort of older, acutely hospitalized patients, showing an independent contribution of dehydration and frailty to increased mortality [31]. 

In contrast, we found that frailty was directly associated with the occurrence of postoperative dehydration, suggesting that this condition-although not evident in the preoperative phase-may quickly develop postoperatively in MPI high-risk older subjects. In these patients, hip fracture surgery may represent an extrinsic stressor unsettling fluid balance and patenting hemodynamic frailty, which determines dehydration. Indeed, the incidence of postoperative acute heart failure exacerbation, a complication possibly associated with hemodynamic frailty, was higher in patients with HD as compared to those with no-HD. These findings are relevant, since most studies analyzing the impact of dehydration on morbidity, mortality and functional outcomes in hip fracture patients have been conducted using the preoperative data. One small study including 38 patients and using a combination of clinical parameters and serum osmolality to diagnose postoperative dehydration reported a higher prevalence (40%), however the relationships with clinical outcomes were not explored [4]. Similar data were reported in another study from the same group including 43 patients in which postoperative HD was diagnosed using a composite score combining urine and plasma parameters [5]. Regardless these discrepancies on the prevalence of postoperative HD which may be influenced by the protocols adopted for hemodynamic management of fluids in the perioperative period, our results underpin the concept that standard approaches to optimize hydration status during hip fracture surgery, that are highly effective in older fit patients, may be inadequate for frail subjects. Importantly, it should be noted that in the present investigation clinicians were unaware about patients’ serum osmolarity, which was instead calculated retrospectively for research aims. We might speculate that the integration of serum osmolarity in the patient’s preoperative assessment could have driven interventions aimed at better correcting preoperative HD and preventing its postoperative persistence or onset.

Postoperative HD was also an independent risk factor for increased hospital LOS along with severity of disease as defined by the Glasgow prognostic score. Based on previous literature [24,31,32], the selected cut offs for moderately-to-severely decreased renal function, GPS poor prognosis and MPI severe risk were, respectively, an eGFR <60 mL/min/1.73 m^2^, a GPS score of 2 and a MPI value of 0.67–1.00. These cut offs have been shown to correlate with adverse clinical outcomes, included prolonged LOS and in-creased mortality. Conversely, a more severe frailty level as described by MPI was predictive of a significantly shorter hospital LOS. Intuitively, while acute conditions have a direct impact on longer hospital LOS, uncomplicated frailty may not require prolonged hospitalization. 

Finally, for the first time to our knowledge, we documented the effect of postoperative HD on short-, medium- and long-term mortality. Our findings demonstrate that all-cause mortality progressively increased over time up to 20% at one year in older hip fracture patients with postoperative HD, being significantly higher than that in patients without HD at 60, 90, 180 and 365 days. The effect of dehydration on mortality was independent of other factors which showed a correlation with mortality in bivariate analysis and were included in the final model, namely frailty, severity of disease and male sex. In contrast, mortality was not associated with postoperative HD at 30 days. This finding is in line with that reported by Hahn in a smaller group of patients [5]. 

We should however acknowledge that the study has some limitations, mainly related to the retrospective design, that should be considered when interpreting the results. First, we enrolled a convenience sample of 599 patients corresponding to the population admitted to the study setting because of a femur fracture over one year ago. Considering the one-year mortality in normo- and dehydrated subgroups, a post-hoc analysis revealed that a sample size of 680 subjects would have been needed to ensure an 80% power with an α-error of 0.05. Second, only a limited number of confounders were considered to adjust multivariable regression models because of the relatively low number of events observed within this study period: other variables not considered in this study could have affected the study outcomes. Moreover, a certain number of data to calculate Glasgow Prognostic Score were missing; the availability of data for the entire population could have led to different results. In addition, measured serum osmolarity was not available for many patients and for this reason calculated osmolarity was used. However, a direct measurement should be warranted. Finally, this study was conducted in a single center, which implies that ward-specific factors (e.g., staffing, nursing skill mix, organizational issues) may have influenced patient outcomes. A multicenter approach would be necessary to improve generalizability of our results. 

Nonetheless, some findings of this study are worth mentioning because of their clinical implications. First, frail patients who are not dehydrated in the preoperative phase may develop dehydration postoperatively; therefore, older subjects scoring at high risk for mortality due to frailty should be strictly monitored even though dehydration is not apparent at admission. Second, the use of standard protocols to optimize the hydration status in the perioperative phase may be less efficient in frail patients, especially in the presence of heart or kidney failure, which impose limits on the possibility of expanding the circulating volume. Third, since postoperative dehydration is a marker of increased morbidity, hospital LOS and mortality, development of individualized approaches to hemodynamic handling in high-risk patients should be considered. The mortality benefit of a precision medicine approach to prevent undesired outcomes in these patients warrants further investigation.

## 5. Conclusions

Older frail patients with hip fracture are prone to developing postoperative hyperosmolar dehydration after surgery, especially when affected by chronic heart or kidney failure. The frailest subjects are particularly exposed to the risk of getting postoperative dehydration, which is associated with an increased likelihood of developing complications and independently predicts prolonged hospital LOS and mid- to long-term mortality. These findings strongly support the concept of systematically screening older patients for frailty and dehydration, and of developing individualized protocols to manage hydration status in patients at high risk.

## Figures and Tables

**Table 1:** Main baseline social-demographic and clinical characteristics of the overall study population and of subgroups presenting (*n* = 122) or not (*n* = 477) with a preoperative hyperosmolar dehydration (HD) condition.

Variable	*n*	All	no-HD	HD	*p*-Value
Age (years)	599	84.0; 79.0–90.0	85.0; 78.0–90.0	84.0; 80.0–90.0	0.621
Age > 85 years	599	267 (44.6%)	214 (44.9%)	53 (43.4%)	0.778
Sex (male)	599	151 (25.2%)	121 (25.4%)	30 (24.6%)	0.860
Living in a nursing home	598	114 (19.1%)	98 (20.5%)	16 (13.2%)	0.067
Body Mass Index (kg/m^2^)	598	23.1; 20.9–25.7	22.7; 20.8–25.0	24.5; 22.2–28.4	<0.001
Multidimensional Prognostic Index	596	0.50; 0.37–0.63	0.50; 0.36–0.63	0.50; 0.38–0.63	0.335
Main comorbid conditions	599				
Arterial hypertension	599	361 (60.3%)	269 (56.4%)	92 (75.4%)	<0.001
Chronic heart failure	599	95 (15.9%)	68 (14.3%)	27 (22.1%)	0.034
Chronic kidney disease	599	76 (12.7%)	45 (9.4%)	31 (25.4%)	<0.001
Cognitive impairment (severe)	597	143 (23.9%)	119 (24.9%)	24 (20.0%)	0.256
COPD	599	61 (10.2%)	49 (10.3%)	12 (9.8%)	0.887
Diabetes mellitus	599	122 (20.4%)	88 (18.4%)	34 (27.9%)	0.021
Hypertensive heart disease	599	124 (20.7%)	92 (19.3%)	32 (26.2%)	0.091
Ischemic heart disease	599	112 (18.7%)	86 (18.0%)	26 (21.3%)	0.407
Valvular heart disease	599	74 (12.4%)	60 (12.6%)	14 (11.5%)	0.741
Relevant home therapy	599				
Number of medications	599	4.0; 2.0–6.0	4.0; 2.0–6.0	5.0; 2.0–7.0	0.021
Polipharmacy (>4 drugs)	599	266 (44.4%)	199 (41.7%)	67 (54.9%)	0.009
Diuretics	599	190 (31.7%)	130 (27.3%)	60 (49.2%)	<0.001
Anti hypertensives	599	347 (57.9%)	260 (54.5%)	87 (71.3%)	0.001
Neuroleptics/benzodiazepines	599	231 (38.6%)	189 (39.6%)	42 (34.4%)	0.293
Steroids	599	19 (3.2%)	14 (2.9%)	5 (4.1%)	0.513
Preoperative laboratory blood tests	599				
eGFR (mL/min/1.73 m^2^)	599	71.6; 54.9–89.3	75.7; 60.2–93.0	53.9; 38.0–70.6	<0.001
Urea (mmol/L)	599	7.3; 5.8–9.9	6.8; 5.3–8.7	11.2; 8.5–13.5	<0.001
Sodium (mmol/L)	599	138.0; 136.0–140.0	137.0; 135.0–139.0	140.0; 138.0–141.0	<0.001
Potassium (mmol/L)	599	4.0; 3.7–4.3	4.0; 3.7–4.3	4.2; 3.9–4.6	<0.001
Fasting glucose (mmol/L)	599	7.1; 6.3–8.3	7.0; 6.2–8.1	7.9; 6.6–9.7	<0.001
Serum osmolarity (mmol/L)	599	295.0; 290.5–299.3	293.1; 289.6–296.4	303.7; 302.0–305.6	<0.001
Hemoglobin (g/dL)	599	11.9; 10.6–13.2	12.0; 10.7–13.3	11.4; 10.3–12.9	0.007
Total protein (g/dL)	514	6.2; 5.8–6.6	6.2; 5.8–6.6	6.1; 5.8–6.5	0.489
Albumin (g/dL)	538	3.5; 3.2–3.7	3.5; 3.2–3.7	3.5; 3.3–3.7	0.401
C-reactive protein (mg/L)	562	19.9; 6.5–63.2	19.4; 6.2–62.2	21.0; 7.1–78.0	0.126
Lymphocytes (cells × 10^3^/mL)	522	1.1; 0.8–1.5	1.1; 0.8–1.5	1.1; 0.8–1.4	0.247
Glasgow Prognostic Score = 2	516	186 (31.1%)	152 (37.0%)	34 (32.4%)	0.381

Data are presented as median; interquartile range or number (percentage). COPD: chronic obstructive pulmonary disease. eGFR: estimated Glomerular Filtration Rate.

**Table 2:** Postoperative clinical profile of patients presenting (*n* = 99) or not (*n* = 496) a hyperosmolar dehydration (HD) condition after surgery.

Variable	*n*	no-HD	HD	*p*-Value
Age > 85 years	595	206 (41.5%)	58 (58.6%)	0.002
Sex (male)	595	119 (24.0%)	30 (30.3%)	0.186
Surgery after two or more days	595	128 (25.8%)	40 (40.4%)	0.003
Type of surgery	595			0.953
Osteosyntesis		294 (59.3%)	59 (59.6%)	
Arthroplasty		202 (40.7%)	40 (40.4%)	
Blood transfusions	590	207 (42.0%)	52 (53.6%)	0.035
Postoperative complications				
Blood glucose imbalance	590	5 (1.0%)	1 (1.0%)	1.000
Delirium	590	54 (11.0%)	14 (14.4%)	0.327
Electrolyte imbalance	590	101 (20.5%)	29 (29.9%)	0.041
Heart failure exacerbation	590	37 (7.5%)	15 (15.5%)	0.011
Pneumonia	590	11 (2.2%)	8 (8.2%)	0.002
Pressure ulcers	595	0 (0.0%)	2 (2.1%)	0.027
Respiratory failure	590	22 (4.5%)	9 (9.3%)	0.052
Sepsis	590	3 (0.6%)	0 (0.0%)	1.000
Surgical wound infection	590	0 (0.0%)	0 (0.0%)	n.c.
Urinary tract infections	590	19 (3.9%)	5 (5.2%)	0.572

Data are presented as numbers (percentages). n.c.: not comparable.

**Table 3:** Multiple linear regression of study variables on length of hospital stays.

Variables	B	SE (95% CI)	*β*	*p*-Value
Postoperative HD	0.083	0.021 (0.042–0.125)	0.171	<0.001
Glasgow Prognostic Score	0.050	0.017 (0.017–0.082)	0.130	0.003
Multidimensional prognostic index	−0.051	0.019 (−0.088–−0.013)	−0.117	0.008
Sex (male)	0.044	0.018 (0.008–0.080)	0.103	0.018

Predictors included in the regression model: sex, postoperative dehydration, eGFR <60, MPI-severe risk (0.67–1.00), GPS-poor prognosis (2); the predictor not reported in the table was excluded from the final model. SE: standard error. CI: confidence interval. HD: hyperosmolar dehydration.

**Table 4:** Variables predictive for all-cause mortality in bivariate and multivariate survival analyses.

Dependent Variable	Predictor	Unadjusted Risk ^a^ χ^2^; *p*-Value	Adjusted Risk ^b^ HR (95% CI); *p*-Value
30-day mortality	Postoperative HD	6.510; 0.011	n.s.
	MPI-severe risk		4.840 (1.896–12.360); 0.001
	GPS-poor prognosis		4.298 (1.539–12.000); 0.005
60-day mortaility	Postoperative HD	15.429; <0.001	3.084 (1.624–5.854); 0.001
	MPI-severe risk		3.322 (1.760–6.272); 0.001
	GPS-poor prognosis		3.312 (1.720–6.377); <0.001
	Sex		2.269 (1.188–4.333); 0.013
90-days mortality	Postoperative HD	17.015; <0.001	3.155 (1.723–5.775); <0.001
	MPI-severe risk		3.267 (1.795–5.945); <0.001
	GPS-poor prognosis		2.997 (1.632–5.503); <0.001
	Sex		2.529 (1.384–4.623); 0.003
180-days mortality	Postoperative HD	12.339; <0.001	2.392 (1.429–4.002); 0.001
	MPI-severe risk		3.646 (2.244–5.926); <0.001
	GPS-poor prognosis		2.689 (1.652–4.379); <0.001
	Sex		2.016 (1.212–3.356); 0.007
365-days mortality	Postoperative HD	5.522; 0.019	1.655 (1.056–2.594); 0.028
	MPI-severe risk		3.502 (2.365–5.186); <0.001
	GPS-poor prognosis		1.818 (1.234–2.678); 0.002
	Sex		2.159 (1.437–3.244); <0.001

Predictors included in the regression models: age > 85, sex (male); postoperative hyperosmolar dehydration (HD); eGFR < 60; MPI-severe risk (0.67–1.00); GPS-poor prognosis (2); the predictors not reported in the table were excluded from the respective final models. ^a^: Log-Rank Mantel-Cox test. ^b^: multivariable forward stepwise Cox regression analysis. CI: confidence interval. MPI: Multidimensional Prognostic Index. GPS: Glasgow Prognostic Score.

## Data Availability

The data presented in this study are available on request from the corresponding author. The data are not publicly available due to privacy and ethical reasons.

## References

[1] Khan M.A., Hossain F.S., Dashti Z., Muthukumar N. (2012). Causes and predictors of early re-admission after surgery for a fracture of the hip. J. Bone Jt. Surg..

[2] Sheehan K.J., Guerrero E.M., Tainter D., Dial B., Milton-Cole R., Blair J.A., Alexander J., Swamy P., Kuramoto L., Guy P. (2019). Prognostic factors of in-hospital complications after hip fracture surgery: A scoping review. Osteoporos. Int..

[3] Sheehan K.J., Williamson L., Alexander J., Filliter C., Sobolev B., Guy P., Bearne L.M., Sackley C. (2018). Prognostic factors of functional outcome after hip fracture surgery: A systematic review. Age Ageing.

[4] Ekman L., Johnson P., Hahn R.G. (2020). Signs of dehydration after hip fracture surgery: An observational descriptive study. Medicina.

[5] Hahn R.G. (2015). Renal injury during hip fracture surgery: An exploratory study. Anaesthesiol. Intensive Ther..

[6] Ylienvaara S., Elisson O., Berg K., Zdolsek J.H., Krook H., Hahn R.G. (2014). Preoperative urine-specific weight and the incidence of complications after hip fracture surgery. A prospective, observational study. Eur. J. Anaesthesiol..

[7] Adunsky A., Mizrahi E.H., Kaplan A., Purits E., Waitzman A., Arad M. (2011). Elevated blood urea, independent of glomerular filtration rate (GFR), confers increased risk of adverse functional outcome in elderly hip fracture patients. Arch. Gerontol. Geriatr..

[8] Docherty N.G., Delles C., D’Haese P., Layton A.T., Martínez-Salgado C., Vervaet B.A., López-Hernández F.J. (2021). Haemodynamic frailty—A risk factor for acute kidney injury in the elderly. Ageing Res. Rev..

[9] Xu B.Y., Yan S., Low L.L., Vasanwala F.F., Low S.G. (2019). Predictors of poor functional outcomes and mortality in patients with hip fracture: A systematic review. BMC Musculoskelet. Disord..

[10] Collin C., Bimou C., Mabit C., Tchalla A., Charissoux J.L., Marcheix P.S. (2020). Orthogeriatric assessment of patients over 75 years of age with a proximal femur fracture: Predictors of 6-month mortality. Orthop. Traumatol. Surg. Res..

[11] Pilotto A., Custodero C., Maggi S., Polidori M.C., Veronese N., Ferrucci L. (2020). A multidimensional approach to frailty in older people. Ageing Res. Rev..

[12] Pioli G., Bendini C., Pignedoli P., Giusti A., Marsh D. (2018). Orthogeriatric co-management—managing frailty as well as fragility. Injury.

[13] Katsoulis M., Benetou V., Karapetyan T., Feskanich D., Grodstein F., Pettersson-Kymmer U., Eriksson S., Wilsgaard T., Jørgensen L., Ahmed L.A. (2017). Excess mortality after hip fracture in elderly persons from Europe and the USA: The CHANCES project. J. Intern. Med..

[14] Van Heghe A., Mordant G., Dupont J., Dejaeger M., Laurent M.R., Gielen E. (2022). Effects of orthogeriatric care models on outcomes of hip fracture patients: A systematic review and meta-analysis. Calcif. Tissue Int..

[15] Kusen J.Q., Schafroth B., Poblete B., van der Vet P.C.R., Link B.C., Wijdicks F.J.G., Babst R.H., Beeres F.J.P. (2019). The implementation of a Geriatric Fracture Centre for hip fractures to reduce mortality and morbidity: An observational study. Arch. Orthop. Trauma Surg..

[16] Prestmo A., Hagen G., Sletvold O., Helbostad J.L., Thingstad P., Taraldsen K., Lydersen S., Halsteinli V., Saltnes T., Lamb S.E. (2015). Comprehensive geriatric care for patients with hip fractures: A prospective, randomised, controlled trial. Lancet.

[17] Salvador-Marín J., Ferrández-Martínez F.J., Lawton C.D., Orozco-Beltrán D., Martínez-López J.F., Kelly B.T., Marzo-Campos J.C. (2021). Efficacy of a multidisciplinary care protocol for the treatment of operated hip fracture patients. Sci. Rep..

[18] Khajuria A., Krahn J. (2005). Osmolality revisited—Deriving and validating the best formula for calculated osmolality. Clin. Biochem..

[19] Hooper L., Abdelhamid A., Ali A., Bunn D.K., Jennings A., John W.G., Kerry S., Lindner G., Pfortmueller C.A., Sjöstrand F. (2015). Diagnostic accuracy of calculated serum osmolarity to predict dehydration in older people: Adding value to pathology laboratory reports. BMJ Open.

[20] Munk T., Bech C.B., Klausen T.W., Rønholt F., Suetta C., Knudsen A.W. (2021). Accuracy of the calculated serum osmolarity to screen for hyperosmolar dehydration in older hospitalised medical patients. Clin. Nutr..

[21] Volkert D., Beck A.M., Cederholm T., Cruz-Jentoft A., Goisser S., Hooper L., Kiesswetter E., Maggio M., Raynaud-Simon A., Sieber C.C. (2019). ESPEN guideline on clinical nutrition and hydration in geriatrics. Clin. Nutr..

[22] Levey A.S., Stevens L.A., Schmid C.H., Zhang Y.L., Castro A.F., Feldman H.I., Kusek J.W., Eggers P., Van Lente F., Greene T. (2009). A new equation to estimate glomerular filtration rate. Ann. Intern. Med..

[23] Warnier R.M., van Rossum E., van Velthuijsen E., Mulder W.J., Schols J.M., Kempen G.I. (2016). Validity, Reliability and feasibility of tools to identify frail older patients in inpatient hospital care: A systematic review. J. Nutr. Health Aging.

[24] Pilotto A., Ferrucci L., Franceschi M., D’Ambrosio L.P., Scarcelli C., Cascavilla L., Paris F., Placentino G., Seripa D., Dallapiccola B. (2008). Development and validation of a multidimensional prognostic index for one-year mortality from comprehensive geriatric assessment in hospitalized older patients. Rejuvenation Res..

[25] Budzynski J., Tojek K., Czerniak B., Banaszkiewicz Z. (2016). Scores of nutritional risk and parameters of nutritional status assessment as predictors of in-hospital mortality and readmissions in the general hospital population. Clin. Nutr..

[26] Kusen J.Q., van der Vet P.C.R., Wijdicks F.J.G., Link B.C., Poblete B., van der Velde D., Babst R., Beeres F.J.P. (2021). Does preoperative hemodynamic preconditioning improve morbidity and mortality after traumatic hip fracture in geriatric patients? A retrospective cohort study. Arch. Orthop. Trauma Surg..

[27] Hooper L., Abdelhamid A., Attreed N.J., Campbell W.W., Channell A.M., Chassagne P., Culp K.R., Fletcher S.J., Fortes M.B., Fuller N. (2015). Clinical symptoms, signs and tests for identification of impending and current water-loss dehydration in older people. Cochrane Database Syst. Rev..

[28] Löffel L.M., Engel D.A., Beilstein C.M., Hahn R.G., Furrer M.A., Wuethrich P.Y. (2021). Dehydration before major urological surgery and the perioperative pattern of plasma creatinine: A prospective cohort series. J. Clin. Med..

[29] Hooper L., Bunn D.K., Downing A., Jimoh F.O., Groves J., Free C., Cowap V., Potter J.F., Hunter P.R., Shepstone L. (2016). Which Frail Older People Are Dehydrated? The UK DRIE Study. J. Gerontology. Ser. A Biol. Sci. Med. Sci..

[30] Lancaster K.J., Smiciklas-Wright H., Heller D.A., Ahern F.M., Jensen G. (2003). Dehydration in black and white older adults using diuretics. Ann. Epidemiol..

[31] Zanetti M., Marzaro G., De Colle P., Toigo G., Bianchini D., Nastri M., Suriano C., Barazzoni R., Sanson G. (2021). Predictors of short- and long-term mortality among acutely admitted older patients: Role of inflammation and frailty. Aging Clin. Exp. Res..

[32] Van Pottelbergh G., Den Elzen W.P., Degryse J., Gussekloo J. (2013). Prediction of mortality and functional decline by changes in eGFR in the very elderly: The Leiden 85-plus study. BMC Geriatr..

